# High-throughput sequencing analysis of genes encoding the B-lymphocyte receptor heavy-chain CDR3 in renal and peripheral blood of IgA nephropathy

**DOI:** 10.1042/BSR20190482

**Published:** 2019-10-15

**Authors:** Dapeng Chen, Zheng Zhang, Yue Yang, Quan Hong, Wenge Li, Li Zhuo

**Affiliations:** 1 Department of Nephrology, China-Japan Friendship Hospital, Beijing 100029, P.R. China; 2 Department of Nephrology, PLA General Hospital, Institute of Nephrology, Beijing Key Laboratory of Kidney Disease, State Key Laboratory of Kidney Diseases, National Clinical Research Center for Kidney Diseases, Beijing 100853, P.R. China

**Keywords:** B lymphocyte receptor, CDR3, High-throughput sequencing, IgA nephropathy

## Abstract

**Aim:** IgA nephropathy (IgAN) is one of the most common chronic glomerulonephritis. Its etiology and pathogenesis remain unclear. We thus explored the immune repertoire of the B-cell receptor (BCR) and the heavy-chain complementarity-determining region 3 (CDR3) in renal tissue and peripheral blood of IgAN patients. **Method**: Total RNAs extracted from renal tissues and peripheral blood of patients and peripheral blood of healthy controls (HCs) were analyzed via high-throughput multiplex PCR sequencing. We amplified and sequenced BCR heavy-chain CDR3 regions to explore repertoire diversity, V/J gene family distribution, CDR3 lengths, BCR heavy-chain variants, consistency between tissue and peripheral blood data, and clones ‘shared’ by these bodily compartments. **Results:** We identified the renal tissue and peripheral blood BCR heavy-chain CDR3 immune repertoires of 15 IgAN patients. *Top1* could be more readily cloned from peripheral blood of patients than from controls (*P<0.05*), the average CDR3 length was significantly shorter in patients than in HCs (*P<0.05*), the variant frequency of the gene encoding the BCR heavy chain was higher in patients than in HCs (*P<0.05*), and the BCR variant frequency was highest in IgAN kidney tissue. Preliminary screening for ‘shared’ clones showed that, in at least 13 patients, the ‘ALYFHNSAY’, ‘ARWGPMYYYMDV’, ‘ARDQGALNA’, and ‘ARVDNPADF’ CDR3 sequences were evident in peripheral blood samples from patients, but not HCs. **Conclusions:** We found that the ‘ALYFHNSAY’, ‘ARWGPMYYYMDV’, ‘ARDQGALNA’, and ‘ARVDNPADF’ clonal sequences may be useful for noninvasive diagnosis and treatment planning in IgAN.

## Introduction

IgA nephropathy (IgAN) was first described by Berger in 1968, the condition is also known as Berger’s disease. IgAN is one of the most common forms of chronic glomerulonephritis, accounting for primary disease in 45%; 5–20% and 30-40% of IgAN developing in 10 and 20 years before end-stage renal failure (ESRD). However, the diagnosis, etiology, and pathogenesis of IgAN remain unclear, although serum IgA levels are high in IgAN patients. The pathological characteristics of IgAN include glomerular deposition of IgA or IgA-based immunoglobulins, glomerular mesangial cell proliferation, and extracellular matrix accumulation. During IgAN onset and progression, removal of glycosylated IgA1 from blood triggers the expression of glycan-specific IgG and IgA autoantibodies, stimulates antigen–antibody reactions, causes immune complexes to be deposited in the kidney, and induces nephritis [1]. Most studies suggest that IgAN may be a systemic autoimmune disease whose pathogenesis is closely linked to humoral immunity [2]. The B-cell receptor (BCR), a surface membrane immunoglobulin, is an important component of the B-lymphocyte recognition antigen, and exhibits antigen-binding specificity. Stimulation by a specific antigen changes the selective distribution of the BCR spectrum, which often indicates clonal B-cell proliferation and functional changes in the corresponding B cells [3,4]. We performed multiplex PCR and high-throughput sequencing to determine BCR expression in renal biopsy tissue and the peripheral blood of patients and the peripheral blood of healthy controls (HCs). We obtained BCR *CDR3* gene sequences and subjected them to bioinformatics analysis. We combined this information with clinical data and the features of disease in both renal tissue and peripheral blood. We explored BCR heavy-chain repertoire diversity in terms of the complementarity-determining region 3 (CDR3) sequences. We sought to find diagnostic markers of IgAN non-invasiveness and markers facilitating early diagnosis, detection, and treatment.

## Materials and methods

### Study subjects

Fifteen IgAN patients aged 15–52 years were diagnosed, as either in- or out-patients, at the China-Japan Friendship Hospital (Table 1). Their clinical manifestations and immune pathologies were recorded, and all underwent standard renal biopsies to diagnose IgAN. No patient had a serious heart disease or any disease of the lung, liver, kidney, or other important organ. We enrolled 17 healthy volunteers matching with the patients in terms of gender and age. Table 2 lists the clinical data of the 15 patients. The selection criteria for HCs were: (1) age and gender matched; (2) no apparent self-perceived discomfort and abnormality in the follow-up health checks; (3) no biological relationship with each other; (4) no medical history of autoimmune disorders, cancers, infectious diseases, liver diseases, allergy, and diabetes; and (5) no family history of autoimmune diseases.

**Table 1:** Fifteen patients with IgAN tissue and peripheral blood and 17 cases of HCs peripheral blood of BCR heavy chain

ID	Type	Raw reads (bp)	Used reads (bp)	Total CDR3	Unique CDR3	Shannon	Top1	Aver_len (bp)	HEC_rate (0.1)
1	PBMC	6197670	4981846	3770878	64424	10.42	3.24	13.52	0.0028
1	Tissue	6994896	5665933	4225678	38569	8.46	16.94	13.93	0.0028
2	PBMC	6661422	5401001	3730337	250882	14.00	2.13	13.77	0.00035
2	Tissue	5882260	4844849	3651246	38297	9.64	3.56	14.08	0.0044
3	PBMC	5975953	4847723	3462896	172685	13.85	1.36	13.82	0.00054
3	Tissue	7038299	5615897	4129514	41979	9.77	6.19	14.04	0.0046
5	PBMC	8986796	8366049	7164395	112378	11.90	1.58	13.76	0.0015
5	Tissue	10288726	9451829	8379340	33561	8.65	4.11	13.68	0.0055
7	PBMC	10088389	9318450	7968854	188499	12.53	2.91	13.44	0.00062
7	Tissue	8731756	8018680	6980292	54094	10.25	3.42	13.70	0.0028
10	PBMC	8988276	8344537	7177895	134124	10.08	7.02	14.26	0.00061
10	Tissue	8347783	7734317	6712263	44847	9.96	5.08	13.67	0.0037
16	PBMC	7418635	5830378	4362275	50515	11.20	3.71	13.73	0.0026
16	Tissue	7634461	6208034	4859600	33122	8.83	3.94	14.11	0.0052
17	PBMC	5492068	4370657	3294824	41114	11.13	3.26	13.75	0.0029
17	Tissue	7048329	5734522	4438939	29319	8.24	7.82	13.16	0.0033
19	PBMC	6176691	5032944	3519990	111090	12.62	2.68	13.67	0.00089
19	Tissue	6897621	5461962	4202091	31045	8.65	5.89	13.41	0.0042
24	PBMC	5332917	4372554	3186186	100055	11.18	6.35	13.52	0.0013
24	Tissue	4974765	4144479	3186193	27876	8.02	8.15	14.35	0.0041
27	PBMC	5942925	4812419	3630193	45638	11.46	1.08	13.94	0.0032
27	Tissue	5856264	4693461	3419259	22136	7.38	10.56	14.01	0.0054
28	PBMC	5985358	4985209	3483879	195563	14.06	2.53	13.96	0.00032
28	Tissue	5592825	4442886	3463912	15950	6.19	18.26	14.33	0.0050
34	PBMC	5470444	4544772	3317802	117799	13.29	0.88	13.89	0.00091
34	Tissue	5888465	4726427	3719951	23161	7.37	9.12	13.73	0.0043
35	PBMC	5427527	4505080	3434118	47153	10.28	4.06	13.39	0.0039
35	Tissue	6550555	5279955	4061130	17446	7.16	6.72	13.87	0.0060
40	PBMC	5892200	4788514	3475220	55378	9.58	7.06	13.76	0.0025
40	Tissue	5833090	4635760	3407858	11767	4.98	28.72	13.73	0.0048
HC1	PBMC	11745589	10289027	9395964	87999	10.55	4.60	14.25	0.0018
HC2	PBMC	10081053	8057309	6180775	65866	10.93	4.10	14.59	0.0016
HC3	PBMC	7602175	6350154	4941667	92726	12.44	3.19	14.49	0.0015
HC4	PBMC	5850348	4759624	3440571	54575	12.13	1.22	13.33	0.0022
HC5	PBMC	12463042	10929066	9758191	142207	13.68	0.82	15.08	0.00076
HC6	PBMC	8503463	7540962	6532996	199646	13.96	0.83	15.08	0.00062
HC7	PBMC	8694452	7662936	6897677	101015	11.73	3.32	14.13	0.0016
HC8	PBMC	8329333	7360870	6575230	116481	12.11	2.41	14.49	0.0010
HC9	PBMC	8729682	7765847	6903670	93028	11.16	2.14	15.91	0.0020
HC10	PBMC	8017954	7096730	6032853	300859	15.12	0.95	14.76	0.00022
HC11	PBMC	7412442	6582725	5571827	308079	14.40	1.25	14.73	0.00021
HC12	PBMC	7535727	6586088	6086925	70024	11.08	2.62	15.17	0.0018
HC13	PBMC	8313829	7395497	6854417	86855	11.64	2.79	15.11	0.0015
HC14	PBMC	7942305	7029063	6469675	72077	11.30	1.78	15.12	0.0020
HC15	PBMC	9074623	7871457	7151031	225242	15.21	0.66	15.20	0.00018
HC16	PBMC	7509272	6622047	6029444	167743	14.01	0.92	14.93	0.00052
HC17	PBMC	8209623	7254345	6452053	372693	16.26	1.33	14.43	0.000060

ID, sample number; PBMC, peripheral blood mononuclear cell; Type, sample types; Raw reads, the sequencing of the original data; Used reads, after filtering is used for analysis of the number of high quality reads; Total CDR3, detect all CDR3 containing the correct reading frame number; Unique CDR3, remove duplicate CDR3 clone number; Shannon Index, H (x) = −sum [P (x) log2 (P (x))], in which P (x) on behalf of cloning frequency, H value, the greater the diversity, the better; Top1, the highest proportion in total clone; Aver_len, the average length of CDR3; HEC_rate, highly amplified clones in the proportion of all the clones.

**Table 2:** Clinical data of 15 patients with IgAN of China-Japan friendship hospital

ID	gender	Age (years)	Lee’s pathology hierarchical	CR (umol/l)	Urea (mmol/l)	eGFR (ml/min/1.73m^2^)	24-h UprV (g)	UOsm (mOsm/kg)
17	Female	29	I	58	4	119	7.81	964
35	Male	15	I	152	12.86	58	6.3	772
24	Female	25	II	—	—	—	4.8	—
1	Male	27	III	212	6.84	37.3	2.63	446
3	Female	29	III	132	8.6	60	—	574
5	Male	52	III	123	6.87	56	3.5	637
10	Female	31	III	64.2	5.33	101	1.44	983
40	Female	30	III	54	5.77	110	1.5	770
2	Male	31	IV	108	3.94	78	5.1	355
7	Male	21	IV	117	5.2	94.5	6.57	—
16	Female	43	IV	64	3.39	102	1.96	—
19	Male	51	IV	101	6.43	65.9	1.06	700
27	Male	42	IV	124	5.98	60	2.1	720
34	Male	30	IV	79	8.69	132	1.34	900
28	Male	38	V	97	6.29	96.65	3.04	694

We obtained kidney tissue and peripheral blood samples using standard procedures. We performed renal biopsies on the 15 IgAN patients and also took peripheral blood samples; we collected blood samples only from the 17 healthy volunteers. Renal biopsy tissue was examined microscopically, placed into RNAlater (QIAGEN) collection tubes, and stored at −80°C. Ficoll-Paque PREMIUM (GE Healthcare) was used to purify peripheral blood mononuclear cells (PBMCs) from peripheral blood samples. These were mixed with TRIzol (Invitrogen) in collection tubes and stored at −80°C.

### Preparation of immune system libraries

The TRIzol method was used to extract total RNA from 15 tissue samples and 32 PBMCs. We employed an Agilent 2100 platform (Agilent Technologies) to perform quality control [8]. After addition of DNase I, we mixed 200 ng of total RNA with 20 ng of each of six random primers, 1 μl of dNTP mixture (all 10 mM), reverse transcription-DEPC, and H_2_O to final volumes of 12 μl. All tubes were incubated at 65°C for 5 min and then placed on ice. After centrifugation, each pellet was incubated with 4 μl of 5× first-strand buffer, 1 μl DTT (0.1 M), and 1 μl RNaseOUT (40 units/μl) (SuperScript® Retrovirus Kit, Invitrogen). After mixing, all tubes were placed at room temperature for 2 min. Next, 1 μl SuperScript™ II RT (Invitrogen) was added, the solutions were mixed, and reverse transcription proceeded (after pretreatment at 70°C for 15 min) for 50 min at 42°C. All samples were then subjected to PCR amplification (Multiplex PCR Kit, QIAGEN); the β forward primer was Vβ (0.2 μΜ), and the β reverse primer was Jβ (both 0.2 μΜ). The PCR protocol was 95°C for 15 min, followed by 30 cycles at 94°C for 30 s, 60°C for 90 s, 72°C for 30 s, 72°C for 5 min, and a drop to 12°C. A QIAquick Gel Extraction (QIAGEN) Kit was used to purify amplification products of 100–200 bp.

PCR amplification was performed using 12 V-area specific primers and 1 J-area degenerate primer. We avoided primer dimers, sought to reduce amplification, and aimed to maximally cover the BCR heavy-chain gene family and J genes and the V family area [5].

After purification, the PCR amplification products were used to prepare DNA sequencing libraries (Illumina). Library quality was confirmed, and the libraries were sequenced using an Illumina Hiseq2500 sequencing platform running PE100 chemistry.

### Bioinformatics

The IMGT website (http://www.imgt.org/), which contains information on B-cell V genes and J and D gene sequences, was used to build a reference sequence dataset. The website states that the 2th V genes encode cysteines at the 3′ ends, and that BCR heavy-chain CDR3 proteins have phenylalanines at the 5′ ends of the regional start and stop sequences [6].

We used IMonitor software [7] to process the original reads. This software runs in the R statistical language (version 2.15.3). We used the package ggplot2 (version 0.9.3.1) for data analysis and diagramming. *P*-values were calculated with the aid of the *t* test. A single asterisk (*) indicated *P*≤0.05; two asterisks (**) *P*≤0.01; and three asterisks (***) *P*≤0.001. Values of *P*≤0.05 were deemed to indicate significant differences.

We calculated the Shannon entropies of highly expressed clones and the highest frequencies of library immune system diversities. Shannon entropy is commonly used to evaluate the diversity of immune system libraries. The index considers not only CDR3 types, but also their ratios. The more uniform the CDR3 distribution is, the highter is the Shannon diversity index. A cloning frequency > 0.1% was defined as high; all such clones were identified by their the highly expanded clonal (HEC) ratios.

At the same time, our team participated as a participant in the work of Huang’s team. Currently, Huang et al.’s article has been published [9], although we are concerned about the different immune expression of IgAN patients, the high-throughput detection techniques we use are identical. The publication of Huang et al. [9]also gives us a strong support for this article. I think that the publication of Huang et al. [9] and our work will open a new chapter in the study of IgAN.

## Results

### Detection of BCR heavy-chain CDR3 genes expressed in IgAN patients and HCs

High-throughput sequencing (using an Illumina platform) was performed on peripheral blood samples and renal tissue samples of 15 IgAN patients to obtain libraries of BCR heavy chains. Each library was filtered to obtain high-quality reads (approximately 5.70 Mb) and compared with databases containing 64.98 and 68.84 Mb of BCR CDR3 sequences (1687297 and 463169 different sequences, respectively). We sought to identify lesions of the BCR CDR3 immune repertoire in renal tissue and peripheral blood of patients. We also analyzed 17 peripheral blood samples from HCs, obtaining approximately 98.79 Mb of CDR3 BCR sequences (2016679 different sequences), to establish the CDR3 immune repertoire of healthy blood. The sequencing data are shown in Table 1.

**Figure 1:**
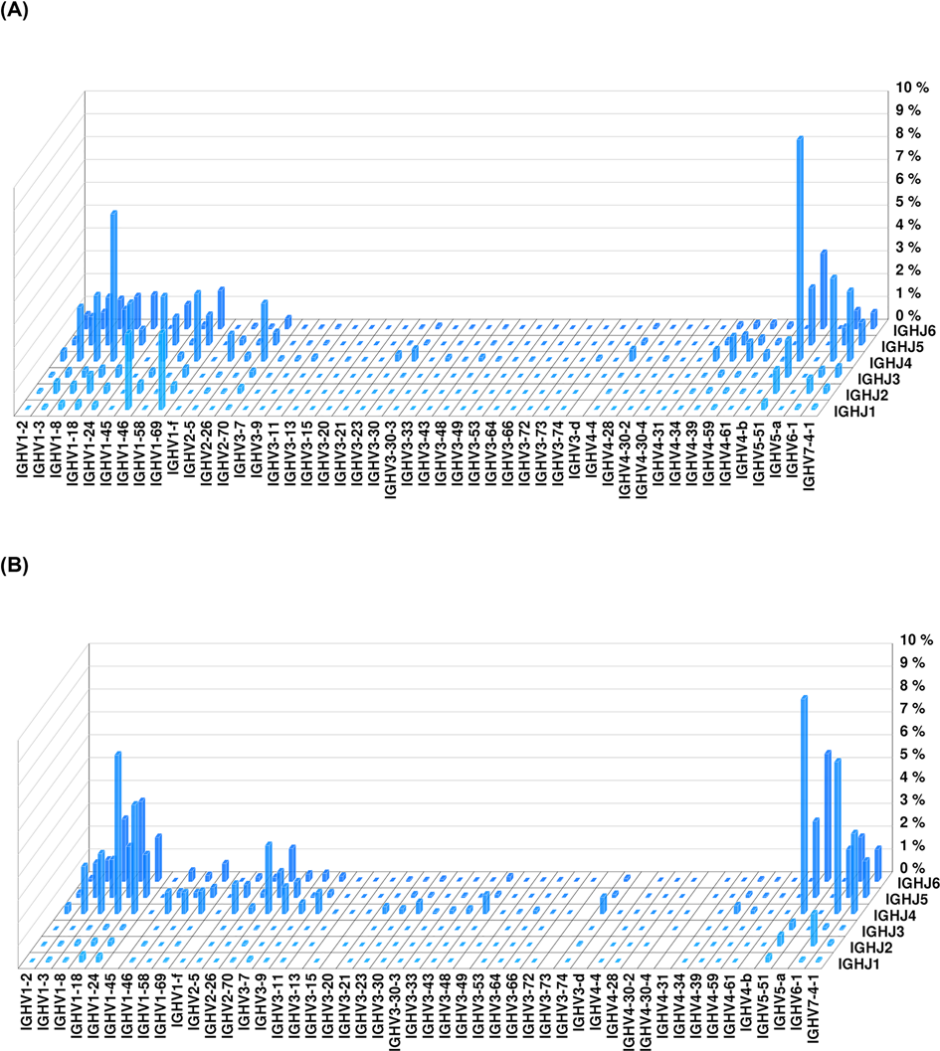
Peripheral blood BCR heavy-chain CDR3 immune library data of an IgAN patient and a healthy volunteer (**A**) Data from IgAN patient #28; (**B**) data from healthy volunteer #15.

Data on the peripheral blood heavy-chain CDR3 BCR immune libraries of patient #28 and HC #15 are shown in three dimensions in Figure 1. The immune repertoire diversity of the patient was lower than that of the healthy volunteer, and some CDR3 sequences were more frequent in the patient.

### Diversity of the BCR heavy-chain immune repertoire in peripheral blood

**Figure 2:**
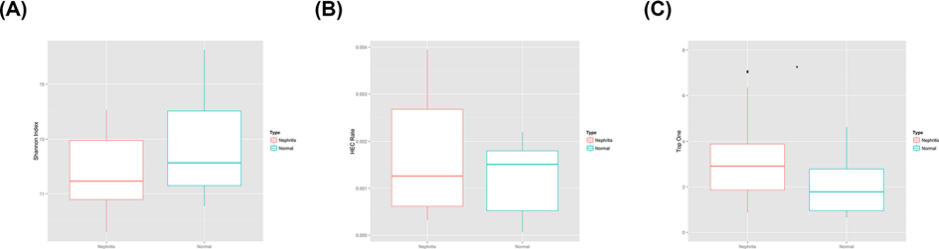
Diversity of BCR heavy-chain groups in the peripheral blood of IgAN patients and HCs (**A**) Shannon diversity index (*P=0.10*); (**B**) HEC ratio (*P=0.17*); (**C**) Top1 clone (*P=0.047*).

The Shannon diversity indices for the peripheral blood BCR heavy-chain immune repertoires of IgAN patients and HCs were 11.84 ± 1.49 and 12.81 ± 1.76, respectively; these values did not differ significantly (Figure 2A). There was thus no significant difference in the peripheral blood CDR3 distribution of BCR heavy-chain immune repertoires between HCs and IgAN patients. We then compared the proportion of highly expressed clones with that of all clones. The HEC ratios of the IgAN patients and controls were 0.0017 ± 0.0012 and 0.0012 ± 0.00074, respectively, which were not significantly different (Figure 2B). Both groups had few highly amplified clones, and there was no significant difference in the extent of amplification. In addition, the *Top1* clone was the most highly expressed in both HCs and IgAN patients. The clonal frequency in IgAN patients (3.32 ± 2.04) was higher than that in HCs (2.05 ±1.22) (Figure 2C).

### Distribution of the V/J gene family of BCR heavy chains in peripheral blood

The distributions of specific V and J subtypes in the peripheral blood of IgAN patients and HCs were evaluated by calculating the proportions of sequences in the V and J gene families.

**Figure 3:**
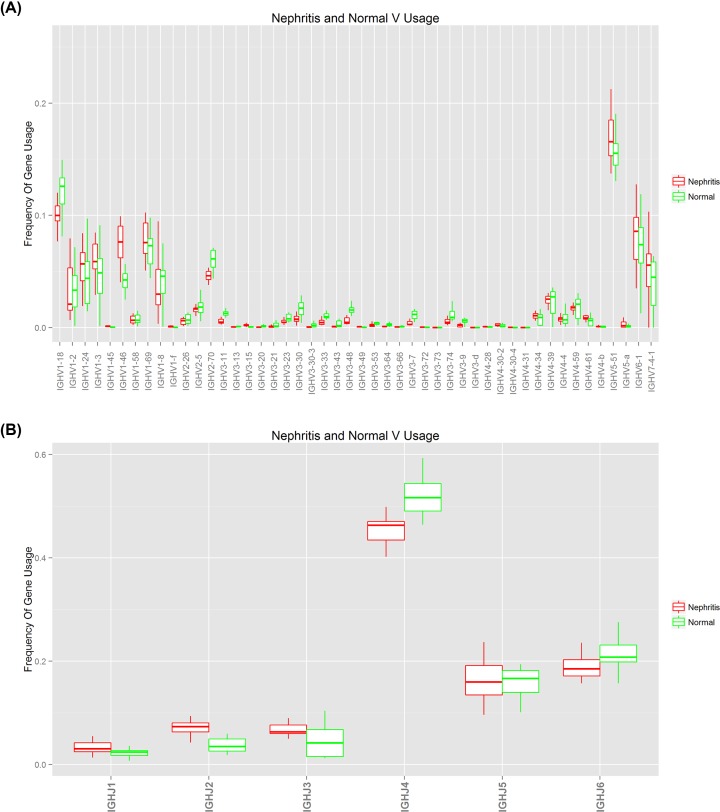
Distribution of V and J gene subtypes among peripheral blood BCR heavy-chains of HCs and IgAN patients (**A**) V gene distribution (*P=0.93*); (**B**) J gene distribution (*P=1.00*).

As shown in Figure 3, 48 V subtypes of 7 V gene families and 6 J genes were expressed in the peripheral blood BCR heavy-chain libraries of both IgAN patients and HCs. The frequencies of V1, V5, V6, V7 and J4, J5, and J6 were higher than others. The two groups did not differ significantly in terms of either V or J gene distribution (Figure 3A,B).

### BCR regional length distribution in CDR3 heavy chains of peripheral blood

**Figure 4:**
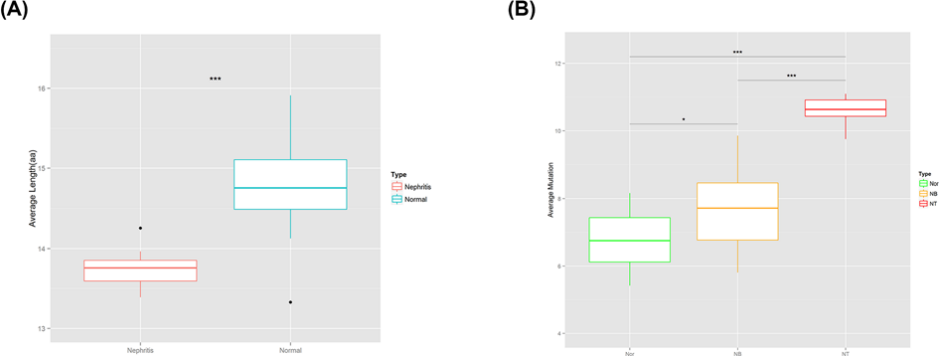
CDR3 lengths and BCR heavy-chain variant frequencies in the peripheral blood of IgAN patients and HCs (**A**) The peripheral blood BCR heavy-chain repertoire in terms of CDR3 length in HCs and IgAN patients (*P=1.02e-06*); (**B**) the peripheral blood IgAN variant frequencies of genes encoding BCR heavy chains in IgAN patients and HCs. Abbreviations: NB, peripheral blood of IgAN patients; Nor, peripheral blood of HCs; NT, tissue of IgAN patients.

The literature suggests that the length of the CDR3 region affects the three-dimensional structure of the CDR3 ring, thus influencing antigen-binding specificity. Therefore, we calculated the CDR3 lengths of the IgH sequences of BCR heavy chains of IgAN patients and HCs. The average CDR3 length in IgAN patients was 13.74 ± 0.22 nt, significantly shorter than that of HCs (14.76 ± 0.57 nt) (Figure 4A).

### Frequency of BCR heavy-chain variants in tissue and peripheral blood

BCR formation should precede gene rearrangement in somatic cells. During an immune response, mature B cells stimulated by antigens during differentiation and development accumulate high levels of somatic variants, increasing BCR diversity. Analysis of variants in genes encoding BCR heavy chains is important in terms of infection, aging, and the development of autoimmune diseases and tumors. We found (Figure 4B) that tissues from patients had an average of 10.64 ± 0.37 IgH variants, significantly more than found in the peripheral blood of both patients (7.71 ± 1.27, *P=1.83e-07*) and controls (6.70 ± 0.88, *P=4.81e-14*). Also, the average number of IgH variants in the peripheral blood of patients was higher than that in controls (Figure 4B, *P=0.016*). BCR variant frequency was obviously higher in patients than controls, and was highest in renal tissue.

### Comparison of BCR heavy-chain groups in tissue and peripheral blood

A correlation study showed that consistency between tissue and peripheral blood data indirectly reflected disease characteristics. We normalized the numbers of BCR heavy-chain CDR3 sequences in patient tissue and peripheral blood samples, selected 10000 sequences, and then calculated Pearson correlation coefficients of patient tissue and peripheral blood libraries with control libraries. Fewer renal tissue BCR heavy chains (and thus CDR3s) were expressed in both tissues and peripheral blood of IgAN patients (Pearson correlation coefficients < 0.2 for all patients).

### Characteristics of tissue and peripheral blood BCR heavy-chain libraries in clonal terms

To further explore changes in the renal and peripheral blood BCR heavy-chain repertoires of IgAN patients, we identified renal BCR heavy chains that ‘shared’ CDR3 sequences in 15 IgAN patients and analyzed their expression levels in the corresponding peripheral blood samples. The results showed that a total of 28268 BCR CDR3 sequences were shared by different IgAN patient tissue B cells. Among them, 14, 13, 12, 10, 9, and 8 patients had ‘common’ sequences of 1, 3, 55, 41, 57, 58, and 118 species. We found that at least 13 patients had the ‘ALYFHNSAY’, ‘ARWGPMYYYMDV’, ‘ARDQGALNA’, and ‘ARVDNPADF’ sequences; we calculated their frequency distributions in tissues and their average frequencies in peripheral blood of the 15 patients mentioned above and of all patients. We found (Table 3) that the ‘common’ four CDR3 sequences were present at high frequencies in peripheral blood. In addition, the ‘shared’ CDR3 sequences of patients differed considerably in both abundance and length. This may be associated with antigenic stimulation during disease progression and development of the immune response.

**Table 3:** IgAN disease characteristics in terms of the four types of BCR heavy-chain CDR3 sequences

CDR3	Freq SN															Average frequency in peripheral blood
	17	35	24	1	3	5	10	40	2	7	16	19	27	34	28	
ALYFHNSAY	3687	24663	2	94793	24	23871	4249	801	27423	0	18	3784	29293	53845	2	4399
ARWGPMYYYMDV	1321	713	2	71095	97034	0	4970	29382	27423	0	41207	1267	29293	12	86739	10058
ARDQGALNA	28268	201	31433	3	86	0	50827	476	110	0	4	15795	6	2	2	5238
ARVDNPADF	2542	49327	31433	23698	3	0	5704	29382	27423	0	10	3248	4	26923	86739	4971

## Discussion

IgAN is the most common primary glomerular disease; both the pathogenesis and the detailed mechanism of progression remain unclear. It is generally believed that large amounts of glycosylation-deficient IgA1 in blood trigger the development of glycan-specific IgG and IgA autoantibodies and the formation of immune complexes. These are abnormally secreted from the mucosa into the blood, ultimately being deposited in the kidney [1]. Thus, IgAN may be a systemic autoimmune disease; the adaptive immune response may be compromised.

Humoral immunity conferred by B cells is closely associated with the formation of specific IgG and IgA autoantibodies, which in turn maybe intimately connected to IgAN development1. The BCR is important in terms of B-cell recognition and antigen binding, thus playing a key role in the humoral immune response. Effective adaptive immunity requires a rich and diverse BCR repertoire2. Adult peripheral blood contains approximately 1–2 × 10^11^ B lymphocytes and 3–9 × 10^9^ BCR heavy-chain CDR3s3. Each B lymphocyte produces a functional BCR heavy-chain CDR3 protein. Therefore, estimation of the diversity of BCR heavy-chain CDR3 sequences allows us to define the extent of amplification of the corresponding B-cell clones. This, in turn, measures the functional status of B cells. Considering that B cell-mediated humoral immunity is closely related to the formation of IgG and IgA autoantibodies specific for polysaccharides, it may be closely related to the formation of IgAN. The reason why we did not focus on the analysis of sIgA and sIgD B cell subsets was due to our current research methodology. We studied the B cell receptor and the CDR3 sequence of the multiple PCR and NGS, which is not included in the C region of the primers. Unfortunately, there no chance for classification analysis. This article is also our team in this area of the first step in the study, we will further enhance our research methods, hope in the follow-up study, can be classified research. Maybe V-C primers can help us with the next experiment.

Previous studies indicated that the BCR was involved in somatic gene rearrangements during an immune response. The BCR undergoes somatic cell variant, thereby greatly increasing the size of the BCR library. We studied the peripheral blood and renal tissues of 15 IgAN patients and the peripheral blood of 15 healthy volunteers in terms of the BCR heavy-chain immune repertoire. We compared the BCR heavy-chain variant frequencies between the groups and found that this was significantly higher in renal tissue than the peripheral blood of both patients and controls. Also, the IgH variant frequency was significantly higher in the peripheral blood of patients than that of controls. In addition, the BCR of patients exhibited a higher variant frequency, suggesting that antigen-specific IgAN reflected sustained activation of the adaptive immune system B-cell differentiation to form proliferating plasma cells and relatively high-frequency variants. The latter may be closely related to the instigation and progression of IgAN. Because of the pathogenesis of IgAN, the BCR spectrum undergoes a selective distribution due to specific antigen stimulation, often leading to clonal proliferation and functional changes in the corresponding B cells. Based on this, we are only tentatively trying to find specific disease markers in B cells that can be seen due to changes induced by specific antigen stimuli. And the primary purpose is to use this as a noninvasive diagnostic marker for IgAN.

To explore the association between the immune responses of renal tissue and peripheral blood, and the progression of IgAN, 15 patients were compared in terms of peripheral blood and renal tissue BCR heavy chains. All Pearson correlation coefficients were <0.2; only weak correlations were thus evident between renal tissue and peripheral blood samples. However, further analysis showed that at least 13 patients had the ‘ALYFHNSAY’, ‘ARWGPMYYYMDV’, ‘ARDQGALNA’, and ‘ARVDNPADF’ sequences, and also four other types of CDR3 clones. The last were present at high frequencies in the peripheral blood of the 15 patients, but not that of the 15 controls. These four CDR3 clones may be characteristic of IgAN, being present at high frequencies in both renal tissue and peripheral blood. They may serve as noninvasive diagnostic markers of IgAN, but further research and clinical verification are required. Before completing the present study, we did not know that the BCR variants were not synchronized in the tissue and in the peripheral blood. According to basic immunology, BCR variants come from somatic high frequency variants in antibody affinity maturation stages, so we have reason to speculate that BCR variants in tissues may be caused by stimulation of renal tissue. Peripheral blood and tissue of the different reasons may be more complex, we want to examine whether the peripheral blood can reflect the characteristics of the lesion and so on. Because kidney tissue is the last point of concern, therefore, should be the kidney tissue variant point as a reference, we look forward to finding the same peripheral blood and kidney tissue mutant phenotype. We also continue to expand the sample size on the basis of existing research, and then to verify the markers have been found, and in the further study, it is possible to find new and more reliable and more accurate markers.

## Conclusions

We sequenced the immune repertoires of renal tissue and peripheral blood of IgAN patients and healthy volunteers. We constructed BCR heavy-chain libraries and found that the heavy-chain variant frequency was significantly higher in patients than in controls. IgAN was characterized by four CDR3 clones that may serve as noninvasive markers of disease. We also discovered four high-variant CDR3 sites. We plan to conduct animal experiments to explore the effects of variants at these four sites and their roles in IgAN.

## Abbreviations

BCR: B-cell receptor
CDR3: complementarity-determining region 3
HC: healthy control
HEC: highly expanded clonal
IgAN: IgA nephropathy
PBMC: peripheral blood mononuclear cell
